# Effect of segmental rim defect morphology on press-fit stability of the cementless acetabular cup: a finite element analysis

**DOI:** 10.3389/fmed.2025.1729549

**Published:** 2025-12-10

**Authors:** Xin Zhang, Kewei Lin, Bolin Feng, Haicong Chen, Huan Zhong, Hanbin Ouyang

**Affiliations:** 1 Department of Orthopedic Center, Joint Surgery, The Affiliated Hospital of Guangdong Medical University, Zhanjiang, China; 2 Department of Orthopaedic Trauma, Heze Municipal Hospital, Heze, China

**Keywords:** bone defect, acetabulum, total hip arthroplasty, finite element analysis, press-fit

## Abstract

**Introduction:**

Segmental rim defect (SRD) of the acetabulum is a common type of osteolysis following primary total hip arthroplasty. Accurate quantitative evaluation of severity and morphology of the SRD can provide critical information to surgeons for decision-making on surgical reconstruction. Therefore, we aim to investigate the effect of morphologic features of SRDs on the press-fit stability of cementless cups.

**Methods:**

A 3D finite element model of Sawbones hemipelvic bone was developed using CT scan. Submodels with varying SRD geometries and locations were created, followed by Boolean reaming and virtual 1-mm press-fit implantation of a cementless cup. The press-fit stability was evaluated by using the push-out and lever-out tests based on quasi-static non-linear finite element analysis. The peak micromotion (PM) levels of all submodels at the bone-cup interface during the gait cycle were also compared.

**Results:**

Despite the depth and width, the defect in the weight-bearing zone exhibited the lowest average push-out load of 2429 ± 294.19 N and lever-out moment of 100 ± 8.40 Nm comparing to those in other zones. The maximal push-out and lever-out loads of the cup decreased as either the width or depth of defect increased. The percentages of strength reduction for push-out and lever-out tests, respectively up to 47.8 and 34.1%, were significantly higher in the weight-bearing zone comparing to those in the other zones. During the gait cycle, all worst-scenario defects resulted in varying levels of increase in interfacial PM comparing to the intact acetabular rim. In the second half of the gait cycle, the defect in the weight-bearing zone produced the highest level of PM.

**Conclusion:**

In revision total hip arthroplasty (r-THA), a large SRD in the weight-bearing zone can result in the utmost loss of press-fit stability and a detrimental interfacial micromotion, which may lead to early loosening of the cup. Thus, an accurate quantitative evaluation of defect morphology is valuable for surgeons to develop a refined plan for reconstruction of initial stability of a cementless cup.

## 1 Introduction

Total hip arthroplasty (THA) has been one of the most successful orthopedic surgeries and gained immense popularity since the last century. It was reported that over 500,000 THAs per year were performed in the United States (1). However, the mid and late term complications of THA are being increasingly predominant over time due to the aging population in most countries. Aseptic loosening, infection, dislocation, periprosthetic fractures, and liner wear are the main indications for revision total hip arthroplasty (r-THA), wherein the aseptic loosening resulting from osteolysis commonly heads the list (2–4). Previous studies have denoted that the aseptic loosening of acetabular component accounts for the majority of all failed primary THAs which necessitate r-THA (5–7). The essence of cup loosening is the loss of long-term stability due to insufficient bony support. Biological reactions induced by wear particles in histiocyte surrounding the cup results in osteolysis and forms varied bone defects (8, 9). Several widely accepted classification systems have described the morphologic features of these defects (10–12). According to the system developed by the American Academy of Orthopedic Surgeons (AAOS) committee on the hip, the cavitary and segmental rim defects (SRD) accounts for the majority of all acetabular defect types in r-THAs. Besides, another similar scenario associated with SRD is commonly seen in developmental dysplasia of the hip which is characterized by the defective weight-bearing segment (13). These SRDs can weaken the clamping effect on cementless cups and pose enormous challenges to surgeons intraoperatively (14, 15).

In an r-THA involving SRD, success instant stability of the cementless cup depends on the press-fit fixation determined by the circumferential stress from the residual rim. In most cases, skillful reaming and using a more oversized cup (e.g., jumbo cup) are thought to be feasible to regain the press-fit stability (16). However, it remains difficult to achieve stable press-fit fixation in those worse cases where additional screw fixation or other reconstructive techniques are needed. These include structural allograft, porous tantalum augments, and various reconstruction cages (17–19). Even though, press-fit fixation in an SRD is still preferable to enhance the osseointegration, ensuring a long-term survivorship of the cup. Prior to customizing a surgical plan, surgeons should accurately evaluate the quality of host bone and morphologic features of the SRD. The corresponding surgical planning would permit surgeons to adjust their strategies intraoperatively to regain the press-fit fixation. There were several studies focusing on the effect of acetabular bone defect on press-fit stability. Dorr et al. (20) investigated the influence of acetabular anatomy on THAs and proposed a morphological classification system of acetabulum that is related to the biomechanics of the rim. Schulze et al. (21) reported their biomechanical test and identified critical level of rim defect that could significantly impair the press-fit stability. Amirouche et al. (22) used a computer simulating method to reveal that extra under-reaming can be an efficient way to strengthen initial stability. Immel et al. (23) established a series of 3D finite element models to identify the determinants of cup primary stability. They suggested that the optimal choice of implant surface roughness and interference fit should be based on the patient’s bone quality. Another probabilistic finite element analysis (24) proposed a similar viewpoint regarding bone quality and further identified the anterior column as the weakest spot of the rim for press-fit stability. However, to our knowledge, the effect of varied morphologic features of an SRD on cup press-fit stability is still unclear. As reported in aforementioned studies, the finite element method is well suited for elucidating such systematic problems with its precise control of involved variables and high efficiency.

In this study, we used finite element modeling to clarify how morphologic features of an SRD affect the press-fit fixation of a cementless cup. Then we attempt to identify the most vulnerable zone of the rim in which an SRD segmental defect could weightily jeopardize the press-fit stability. The clinical significancy of the present study is to provide a critical understanding of the press-fit biomechanics of a defective acetabulum, aiding surgeons in regaining a stabilized cup in r-THA.

## 2 Material and methods

### 2.1 Three-dimensional modeling of hemipelvis

A composite pelvic bone manufactured in form of a standardized human pelvis was used in this study (Sawbones, No. 3405, Pacific Laboratories, United States). With similar mechanical properties to real bones, the composite pelvis can approximately reproduce the press-fit effect on the implanted cementless cup. The left hemipelvis was scanned using a 64-slice spiral CT scanner (slice thickness: 0.625 mm; spacing: 0.5 mm), producing 480 consecutive DICOM cross-sectional images. These images were then imported into the Mimics 14.0 software (Materialise, Belgium). Following the bone segmentation via thresholding and region growing, three-dimensional models were calculated based on the masks generated. The resulting STL model was imported into Geomagic 2013 (3D Systems, United States) and converted into a solid model in STP format through Non-Uniform Rational B-Spline (NURBS) surface fitting. In the Unigraphics NX 12.0 (Siemens PLM Software, United States) software, a 2 mm thick cortical shell was created by performing an inward offset operation on the outer surface of the hemipelvis, dividing the model into distinct cortical and cancellous parts.

### 2.2 Simulation of the acetabular cup implantation

Prior to modeling the cup implantation, a local coordinate system was defined as the orientation reference where the origin was located at the hip rotation center. Based on the 3D measurements of the bony socket, a hemispherical cup was created with an outer diameter of 58 mm and a thickness of 4 mm. As recommended by Lewineck’s study (25), the cup was oriented in the sagittal and coronal planes using an anteversion angle of 15 degrees and an abduction angle of 45 degrees, respectively. A 3D visualized evaluation of the implantation was conducted by two senior surgeons to ensure that the cup could be secured by sufficient bony support. To achieve the press-fit of 1 mm, a simplified model of the reamer with a diameter of 57 mm was created for socket preparation.

### 2.3 Model development for intact acetabulum and segmental rim defect

**FIGURE 1:**
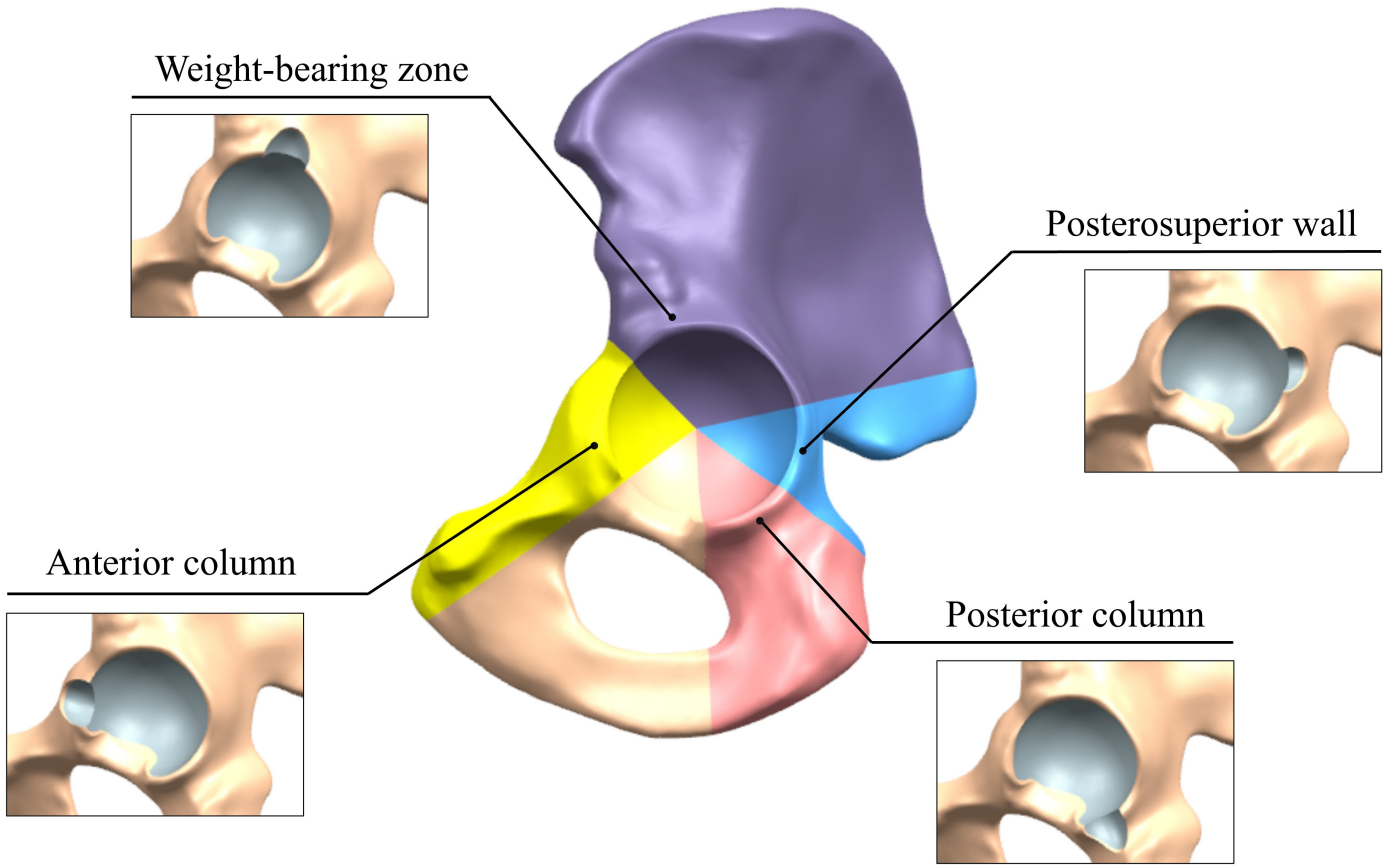
Circle sector partitioning for the SRD of the acetabulum.

A reamed model of the intact acetabular rim was taken as the reference. The SRD models were divided into groups according to the anatomical location and landmarks of the pelvis (26, 27). In the sagittal view, we defined the weight-bearing (WB), anterior column (AC), posterior column (PC), and posterosuperior wall (PSW) zones by a circle sector partition of the rim (Figure 1), where the acetabular notch was excluded. To depict the morphology of defects, we quantified them by their width and depth based on our clinical observation of SRDs and expertise in press-fit implantation. These varying dimensions were combined to design a parametrized ellipsoidal model for SRD generation in each zone. A total of 37 submodels were generated in our study, including one intact rim and 36 SRDs, as shown in Table 1.

**TABLE 1:** Defect size parameters for submodel grouping.

Depth	Width		
	10 mm	15 mm	20 mm
5 mm	D5W10	D5W15	D5W20
10 mm	D10W10	D10W15	D10W20
15 mm	D15W10	D15W15	D15W20
Intact	–	–	–

### 2.4 Development of finite element models

All submodels were imported into the Hypermesh 14.0 (Altair, United States) software for mesh generation. The solid tetrahedral meshing strategy was used for the hemipelvis and acetabular cup. According to the convergence test and consideration of computing time, an element size of 2.5 mm was set in global model. The tetrahedral element (C3D4) numbers of hemipelvis models ranged from 236909 to 302832 while the nodal numbers ranged from 48480 to 69938. And the acetabular cup was comprised of 75008 elements and 17419 nodes. The materials of composite bone and implant were considered homogeneous isotropic and linear elastic (28) (Table 2). A non-linear contact relationship was defined at the bone-cup interface by using a frictional coefficient of 0.6 (23).

**TABLE 2:** Material properties used in finite element models.

Component	Material	Young’s modulus (MPa)	Poisson’s ratio
Sawbones pelvis	Cortical bone	16,700	0.3
	Cancellous bone	155	0.3
Cup	Ti-6Al-4V	110,000	0.3

### 2.5 Biomechanical evaluation

**FIGURE 2:**
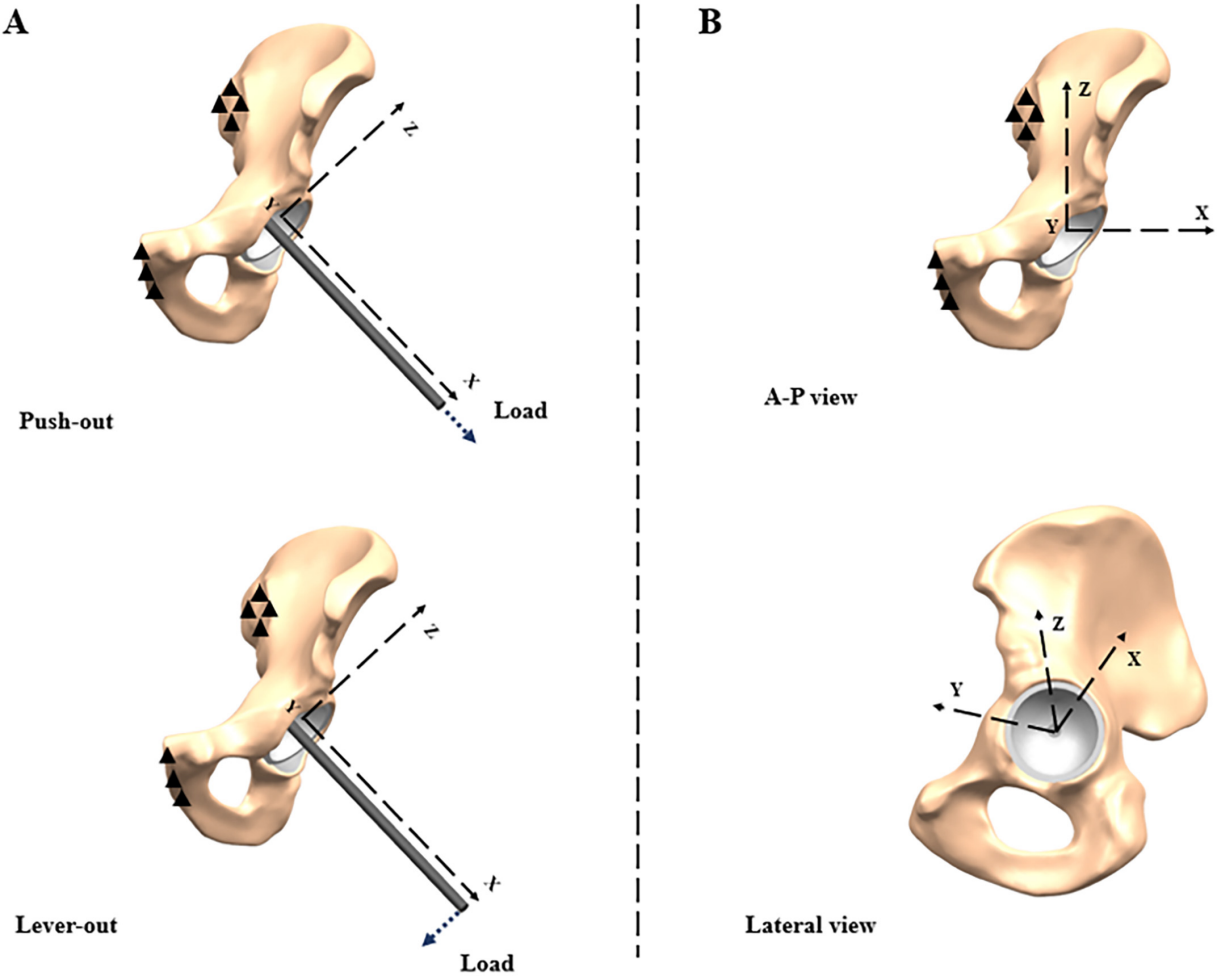
Boundary condition and local coordinate systems for **(A)** load-to-failure tests; **(B)** dynamic loads during gait cycle.

We used two representative biomechanical load-to-failure tests for the strength evaluation of press-fit fixation (29). The sacroiliac and pubic symphysis joint facets were fully constrained as the boundary condition. The analytic process was comprised of three stages: implanting, stabilizing, and loading to failure. In the implantation stage, a predefined mandatory displacement was exerted on the control point centered in the polar screw hole of the cup, inserting the cup into the target position. In the stabilizing stage, the displacement was released to minimize the inertial effect. Eventually, two load-to-failure simulation tests were driven by push-out and lever-out forces, respectively, via a linearly incremental amplitude (Figure 2A). An observed intense uplift of the monitored displacement-time curve denotes a thorough loss of stability. Then, the load at that time-point was recorded as the push-out strength. Meanwhile, another incremental vertical load was exerted on the lateral endpoint of a 20-centimeter-long rigid beam which was rigidly bonded to the polar screw hole of the cup. Similarly, the corresponding moment was recorded as the lever-out strength once an intense uplift of the rotation displacement-time curve was observed.

A simultaneous evaluation of the dynamic variation of peak micromotion (PM) at the bone-cup interface during the gait cycle was also conducted following the stabilizing stage. As shown in Figure 2B, another local coordinate system was created for the load synthesized from three orthogonal components, F_*x*_, F_*y*_, and F_*z*_, respectively. The dynamic loads from a person with a body-weight of 70 kg were adopted as a series of input parameters for our models, according to the study reported by Bergmann et al. (30). The maximal defect of 15 mm in depth and 20 mm in width was used to identify the difference among all zones and compared to the intact group. Finally, all the calculation tasks were submitted to the Abaqus 2016 (Dassault, France) explicit solver.

## 3 Results

### 3.1 Convergence test

The convergence test for model strain energy demonstrated acceptable results, as evidenced by a variation at the level of 5% (Table 3). In the model with further refined mesh sizes, the computational cost increased drastically and even led to the termination of calculation. Based on these results, an element size of 1.5 mm for peri-acetabular region was adopted for all subsequent analyses, as it provides an optimal balance between efficiency and numerical accuracy.

**TABLE 3:** Convergence test results for the intact model.

Peri-acetabular element size (mm)	2.5	2	1.5	1	0.5
Total element count	170,836	241,478	377,043	761,642	2,005,441
Strain energy (mJ)	261.4	242.6	229.7	217.1	NA*
Percentage variation		7.20%	5.30%	5.50%	NA*

*Not available due to calculation termination.

### 3.2 Model validation

We simulated the push-out test in the acetabulum with intact rim following the cup implantation. The cup loosening was determined by a prominent uplift at any point on the displacement-time curve, then the corresponding real-time load was extract as the failure load. In our intact acetabulum model, a maximal load of 3,725 N was calculated from the load-displacement curve in this load-to-failure test. This load level falls in the range from 396.6 ± 7 N to 4118.81 ± 521.82 N as reported in a previous study (29), which can to some extent validate our model.

### 3.3 Failure loads for push-out and lever-out tests

**FIGURE 3:**
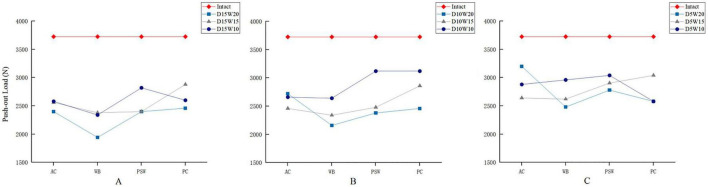
Comparison of failure loads of push-out tests for SRDs varied in depth (**A,** 15 mm; **B,** 10 mm; **C,** 5 mm), width, and location.

Our results showed that the variations in location, depth, and width of SRDs resulted in large difference in the failure loads in push-out tests (Figure 3). Given a specified depth of 15 mm, the failure load for the AC defect was reduced by 35.6% comparing to the intact model (Figure 3A). Moreover, the WB defects presented the most pronounced percentage decreases in failure load among all SRDs, ranging from 36.1 to 47.8%, which were overall proportional to the width of the defect. Besides, a similar downward trend in terms of failure load could be found either in the PSW or PC zone. For the defects of 10 mm depth (Figure 3B), three lines denoted a more typical trend that the failure load declines as the width decreases. A similar trend could also be observed in the defects of 5 mm depth (Figure 3C).

**FIGURE 4:**
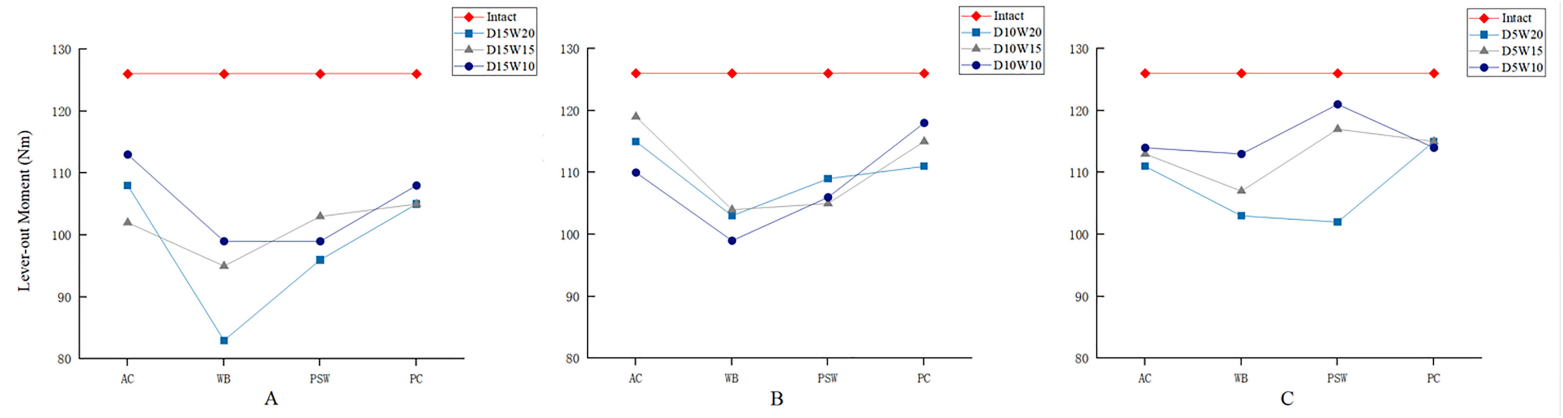
Comparison of failure moments of lever-out tests for SRDs varied in depth (**A**, 15 mm; **B,** 10 mm; **C**, 5 mm), width, and location.

Regarding the lever-out test, the failure moment for the intact model to was approximately 126 Nm. Compared to those results of push-out tests, relatively milder percentage decreases in failure moment, ranging from 4 to 34.1%, were observed in these varied SRDs. Given a specified depth of 15 mm, a declining trend of failure moment associated with the lessening width was observed (Figure 4A). Similarly for both depths of 10 and 5 mm (Figures 4B,C), the results were also consistent with the same width-depending law in variation of the failure moment.

The calculated results considering specified location and depth were listed in Table 4. Covering all depths and widths, SRDs in the WB zone demonstrated the lowest average push-out load of 2429 ± 294.19 N and lever-out moment of 100 ± 8.40 Nm. In contrast, SRDs in the PC zone preserved the highest average push-out load and lever-out moment. On the other hand, a gross descending tendency of average failure load was observed simultaneously in the push-out and lever-out tests as the depth of defect increased. Furthermore, the SRDs with depth of 15 mm led to the lowest average failure loads of 2480 ± 156.17 N and 101 ± 3.36 Nm in the push-out and lever-out tests, respectively.

**TABLE 4:** Average failure loads in the push-out and lever-out tests for specified locations and depths.

Defect type	Average push-out load (N)	Average lever-out moment (Nm)
Location		
AC	2,651 ± 182.24	112 ± 4.80
WB	2,429 ± 294.19	100 ± 8.40
PSW	2,703 ± 292.65	106 ± 8.13
PC	2,731 ± 248.82	112 ± 4.76
Depth		
5 mm	2,789 ± 83.19	112 ± 3.96
10 mm	2,617 ± 238.24	110 ± 1.25
15 mm	2,480 ± 156.17	101 ± 3.38

### 3.4 Peak micromotion at the bone-cup interface

**FIGURE 5:**
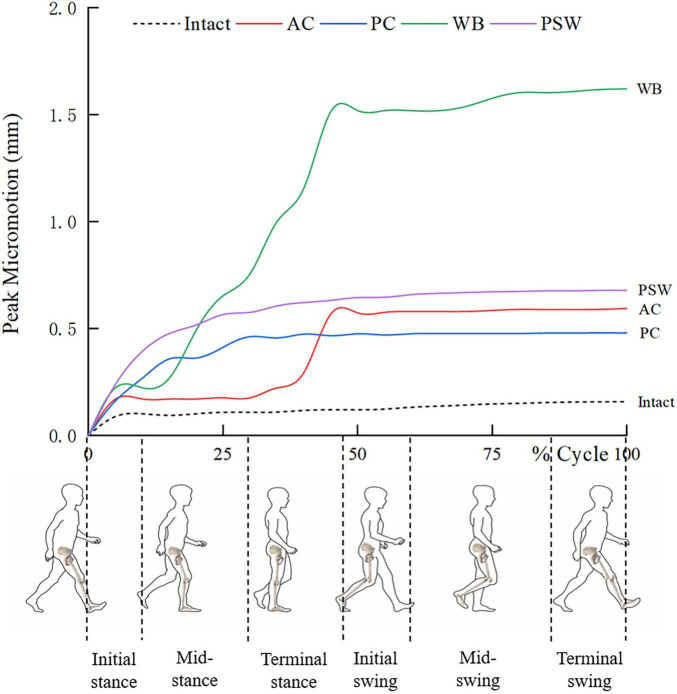
Dynamic variations of peak micromotion during gait cycle for intact rim and SRDs in different locations.

Throughout the whole gait cycle, dynamic variations of PMs at bone-cup interface were recorded for all zones, involving the optimal (intact) and worst (defect of 15 mm × 20 mm) scenarios (Figure 5). In the initial phase (first 10% of the cycle), the PM curve of each SRD model exhibited a distinct uplift varied in scale. Meanwhile, a higher PM could be observed in the PSW model, comparing to those in the others. From 12 to 50% of the cycle, The PM Curve of the WB model began to experience a steep climb then reached a peak far exceeding the heights of the other curves. By comparison, the other model curves presented milder uplifts varied in slope and level. When came to the swing phase, all the PM curves turned flat with diverse levels ranging from 0.45 to 1.62 mm. By the end of the cycle, the WB model produced the highest PM level, while the PC model produced the closest PM level to that of the intact model.

## 4 Discussion

Fundamental knowledge of the press-fit biomechanics is necessary for surgeons to achieve initial and long-term stability of the acetabular cup. In r-THAs, SRDs do bring out various challenges to surgeons since the impact of variability in terms of location and morphologic features on press-fit stability remains unclear. Accurate evaluation of the SRD can help surgeons to achieve success press-fit fixation through making a well-thought-out plan. It is thus critical to uncover the intrinsic connection between biomechanics and defect characteristics. We developed a finite element model of the acetabulum that comprises four functional anatomic zones, including the weight-bearing (WB), anterior column (AC), posterior column (PC), and posterosuperior wall (PSW) zones. SRDs located in these zones with varying morphological parameters were incorporated into our analysis, in accordance with previous biomechanical studies (24). Our simulation results revealed that the loss of press-fit stability increased as the defect size extended either in width or depth. In addition, the SRD located in the WB zone exhibited the worst press-fit stability. This defect type also resulted in the highest PM level which could impede the osseointegration at the bone-cup interface. Overall, our findings suggest that those large SRDs, particularly located in the WB zone, should be of sufficient concern in the surgical planning as they might jeopardize the early biologic fixation of cups.

The most influential segment of the rim on press-fit stability of the cup was found to be in the WB zone. This finding is incompletely consistent with those of previous studies, probably due to the differences in experimental design and subjects. Amirouche et al. previously developed a finite element model to evaluate the cup insertion and fixation in the context of SRDs (24). They revealed that SRDs along the superior or inferior rim have a minor effect on cup insertion and thus may not require additional mechanical augmentation. Similarly, the defect in PC zone, known as the “inferior rim” in their study, results in a comparable decrease in press-fit stability. In contrast, our results demonstrated that the SRD in the WB zone had the most significant impact on press-fit stability. This could be partially attributed to our modified partition method of the acetabulum where the WB zone encompasses both the anterosuperior and posterosuperior segments, as defined in Amirouche’s study. Another potential explanation is that discrepancies in failure test protocol for press-fit evaluation can produce dissimilar results. For the loading method, we employed the push-out and lever-out tests to quantify the fixation strength rather than insertion test. Both of them are representative failure tests and have been widely used in the evaluation of fixation stability for cups (29, 31). In addition, the results exhibited a good consistency between the two tests, which indicated that a common stabilizing mechanism of press-fit fixation in different loading scenarios. Moreover, the “press-fit” refers more to a state than a course, therefore, the load to failure can better reflect the fixation strength.

For any SRDs, the accurate assessment of morphologic features is crucial to elaborating a surgical plan. However, individualized defect types with diverse morphologic features complicates this process so that an effective evaluation system is imperative for surgeons to estimate those potential pitfalls ahead of time. Based on a similar principle to the “barrel theory,” we applied the width and depth in an attempt to grade the SRDs in each zone, which allows for better control of confounding variables and thus can elucidate the effects of those of interest on press-fit stability. Our results revealed that both the two dimensions have considerable impact on the press-fit strength of push-out and lever-out tests. Moreover, the press-fit strength is found to be more sensitive to variation in depth than width. This is also supported by a simulation study on dysplasia acetabular defect reported by Kaku et al. (32). They used the cup-center-edge angle to evaluate the bone-cup interfacial micromotion, revealing an enormous impact of mediolateral defect variations on initial stability. In contrast, several studies have proved that satisfactory press-fit can be achieved even in defects with smaller center-edge angles (21, 33–35). Those defects characterized by large depth are similar to the deep SRDs in our study, and present consistent results that support our findings. However, our results presented a certain oscillation in trends due to some outliers in several tests. This might be attributed to the irregularity of our acetabulum model, which differs from those polymeric foam blocks used in previous studies (29, 31). By comparison, our models considering the pelvic anatomy could reproduce a more realistic local scene for the press-fit evaluation.

Initial stability of the cup determines the level of interfacial micromotion, and mainly depends on the friction force resulting from the circumferential compressive stress at the rim. Maintaining a well-controlled level of interfacial micromotion can facilitate sufficient osteointegration between the bone and cup. Interfacial micromotion of less than 150 μm has been reported as the threshold for bone ingrowth (31). Micromotion exceeding this threshold can induce bone resorption and fibrous tissue ingrowth, potentially leading to early loosening of cups (32). Our physiological loading test results revealed that the SRDs significantly elevate the PM level throughout the gait cycle when compared to the intact model. The high PM level induced by the SRD in the WB zone was particularly pronounced, exceeding the osseointegration threshold. This is consistent with the findings from our failure strength tests, particularly concerning the identification of the most influential segment of the rim. Theoretically, the WB zone constitutes the majority of the iliac bulk and, hence, is regarded as a strut with relative higher structural stiffness (36). Loss of bony buttressing in this zone can lead to a pronounced decrease in interfacial friction for cups. Widmer et al. (37) investigated the load transfer modes of the acetabulum implanted with a cementless cup. They found that the maximum of contact pressure is located at the iliac facet. Janssen et al. (38) found that the PM and maximum contact stress both occur during the mid- and terminal stance phases (25–50%) of the gait cycle, mostly located in the acetabular dome. These findings indirectly support our conclusion that the WB zone is the principal segment of the rim in press-fit fixation, particularly during the latter half of the gait cycle. In contrast, the SRDs in other zones showed a moderate increase in PM. For the cup orientation set in our study, the varied influences of SRDs in these zones on PM might be attributed to the discrepancy in initial bone-cup contact.

The utilization of supplemental screw fixation is a well-established fundamental strategy to achieve stability in cementless acetabular reconstruction, particularly in the presence of segmental rim defects (39). These screws act as anchors and bridge the compromised rim to restore the stability against torsion and pushing loads from daily activities (40). Especially for the early postoperative mobilization, sufficient screw fixation is crucial and reduces interfacial micromotion at the bone-implant interface, facilitating a timely osteointegration (31). Nevertheless, re-reaming the acetabulum would further reduce its bone stock during revision surgery, consequently limiting the available space for screw placement (41). In this scenario, employing an excessive number of screws for enhanced stability will increase the risk of iatrogenic neurovascular injury (42). In contrast, a prudent strategy of screw fixation based on accurate evaluation of press-fit condition might help reduce the risk of such complication. Aimed at identifying the segments that differentially influence the press-fit stability, our work can provide critical insights for guiding the management of cases involving SRDs. Although recognized as a considerable topic of clinical interest, the effect of screw fixation was not included in this study, as it was beyond the scope of our predefined design.

Our study has several limitations. First, a standard composite hemipelvis used in our study cannot incorporate the anatomical diversity (e.g., variations due to gender and age). Other factors such as bone mass, cup size and orientation can affect the final press-fit stability. However, our study aimed to focus on several variables of interest by employing a parameter-controlled design. To fully clarify the underlying press-fit mechanism, further biomechanical tests on large samples of cadaver specimens are necessary. Second, revision-associated pathological changes such as bone sclerosis were not considered in our study, where the interfacial tribology and circumferential stress might be significantly different from those in a healthy population. Nevertheless, the actual mechanical properties of these pathological bone tissues *in vivo* remain unavailable. Third, the geometric features of the SRDs were simplified rather than reproduced as real during the model development. Nonetheless, we considered both depth and width as the primary parameters in our study, as these dimensions might be the most representative for measuring the severity of an SRD. Finally, the use of a single 58-mm cup diameter precludes the generalizability of our findings to diverse ethnic populations, given the known variability in acetabular morphology. This undoubtedly warrants further investigation in future studies. However, the standardized model serves to isolate the influence of the SRD’s location and morphology on press-fit biomechanics.

## 5 Conclusion

A series of SRD models were established to investigate the effect of varied morphologic features on the press-fit stability of the cup. Our simulation results demonstrated that the SRD in the WB zone can significantly impair the press-fit stability of a cementless cup when compared to those in other zones. The degree of impact on press-fit stability is proportional to the depth and width of the defect, which is more pronounced in the former. Additionally, SRDs located in the WB zone may produce much greater interfacial micromotion than those in other zones, which might impede the osseointegration and lead to an early loosening of the cup. Our findings may provide a theoretical basis for surgeons to develop appropriate reconstructive strategies for SRDs in r-THAs. Further cadaveric specimen tests and computational analyses incorporating more relevant factors are needed.

## Data Availability

The raw data supporting the conclusions of this article will be made available by the authors, without undue reservation.
